# ﻿The complete mitochondrial genome of the Mexican-endemic cavefish *Ophisternoninfernale* (Synbranchiformes, Synbranchidae): insights on patterns of selection and implications for synbranchiform phylogenetics

**DOI:** 10.3897/zookeys.1089.78182

**Published:** 2022-03-11

**Authors:** Adán Fernando Mar‐Silva, Jairo Arroyave, Píndaro Díaz-Jaimes

**Affiliations:** 1 Laboratorio de Genética de Organismos Acuáticos, Instituto de Ciencias del Mar y Limnología, Universidad Nacional Autónoma de México, Circuito Exterior S/N, Ciudad Universitaria, 04510 Coyoacán, Mexico City, Mexico Laboratorio de Genética de Organismos Acuáticos, Instituto de Ciencias del Mar y Limnología, Universidad Nacional Autónoma de México Mexico City Mexico; 2 Posgrado en Ciencias del Mar y Limnología, Universidad Nacional Autónoma de México, Mexico City, Mexico Posgrado en Ciencias del Mar y Limnología, Universidad Nacional Autónoma de México Mexico City Mexico; 3 Instituto de Biología, Universidad Nacional Autónoma de México, Circuito Exterior S/N, Ciudad Universitaria, 04510 Coyoacán, Mexico City, Mexico Instituto de Biología, Universidad Nacional Autónoma de México Mexico City Mexico

**Keywords:** Blind swamp eel, karst aquifer, mitogenome, systematics, troglobitic, Yucatan Peninsula

## Abstract

*Ophisternoninfernale* is one of the 200+ troglobitic fish species worldwide, and one of the two cave-dwelling fishes endemic to the karstic aquifer of the Yucatán Peninsula, Mexico. Because of its elusive nature and the relative inaccessibility of its habitat, there is virtually no genetic information on this enigmatic fish. Herein we report the complete mitochondrial genome of *O.infernale*, which overall exhibits a configuration comparable to that of other synbranchiforms as well as of more distantly related teleosts. The K_A_/K_S_ ratio indicates that most mtDNA PCGs in synbranchiforms have evolved under strong purifying selection, preventing major structural and functional protein changes. The few instances of PCGs under positive selection might be related to adaptation to decreased oxygen availability. Phylogenetic analysis of mtDNA comparative data from synbranchiforms and closely related taxa (including the indostomid *Indostomusparadoxus*) corroborate the notion that indostomids are more closely related to synbranchiforms than to gasterosteoids, but without rendering the former paraphyletic. Our phylogenetic results also suggest that New World species of *Ophisternon* might be more closely related to *Synbranchus* than to the remaining *Ophisternon* species. This novel phylogenetic hypothesis, however, should be further tested in the context of a comprehensive systematic study of the group.

## ﻿Introduction

*Ophisternoninfernale* (Synbranchiformes, Synbranchidae), commonly known as the blind swamp eel, is a rare and elusive freshwater teleost fish endemic to the cenotes and submerged caves of the Yucatan Peninsula (YP) in southeastern Mexico. Like most troglobites, *O.infernale* exhibits typical regressive troglomorphic traits associated with life in absolute darkness, such as the absence of both pigmentation and eyes. Besides its endemism and troglomorphism, *O.infernale* is exceptional in that it is one of two fish species that permanently inhabit the dark and oligotrophic subterranean waters of the YP karst aquifer; the other being the Mexican blind brotula (*Typhliaspearsei*) (Arroyave 2020). The relative inaccessibility of its habitat–submerged caves or cenotes well inside dry caves–coupled with its highly cryptic lifestyle–often found burrowed under the sediment or hiding inside tangles of submerged roots and crevices–have made the study of the blind swamp eel particularly challenging, and as a result very little is known about this intriguing fish species. Notably, the total number of occurrence records for *O.infernale* is less than 20 localities throughout its potential range of distribution (Arroyave et al. 2019). By virtue of its rarity, endemism, and restricted geographic distribution, in addition to the current threats faced by its habitat and region, *O.infernale* has recently been categorized as Endangered (EN) (Arroyave et al. 2019). Unsurprisingly, genetic data from *O.infernale* are virtually nonexistent, and this has hampered efforts at establishing its exact phylogenetic placement (Perdices et al. 2005). Besides their importance for phylogenetic and biogeographic research, genomic data are fundamental for addressing other evolutionary lines of inquiry, such as the genetic basis of troglomorphism (Protas and Jeffery 2012). Hence the need for generating genomic information of such a unique, endangered, and understudied species such as *O.infernale*. In order to provide genomic resources potentially informative for future evolutionary studies, here we present the first complete mitochondrial genome of the troglomorphic and YP-endemic *O.infernale*. In addition to sequencing, assembling, and annotating its mitogenome, we present detailed descriptive (genome size and organization, protein-coding genes [PCGs], non-coding regions, and RNAs features) and comparative (patterns of selection on PCGs, phylogenetic) analyses. Leveraging novel mitogenomic data to shed light on the systematics of Synbranchiformes is particularly relevant and timely because of ongoing conflicting hypotheses of relationships regarding the limits and composition of this teleost order that involve the phylogenetic placement of the monogeneric family Indostomidae with respect to synbranchiforms and closely related euteleost lineages (Van Der Laan et al. 2014; Nelson et al. 2016; Betancur-R et al. 2017). Furthermore, the phylogenetic position of the blind swamp eel, *O.infernale*, in the context of the diversification of the family Synbranchidae, has yet to be established (Perdices et al. 2005).

## ﻿Material and methods

### ﻿Sample collection and raw data generation

All methods were carried out in accordance with relevant guidelines and regulations, and the study was carried out in compliance with the ARRIVE guidelines. Sampling of the *O.infernale* individual used to generate the mitogenome presented here was accomplished with the assistance of a professional cave diver who captured the specimen using a custom-made hand net specifically designed for efficient capture and secure storage while cave diving. The sample was collected under collecting permit SGPA/DGVS/05375/19 issued by the Mexican Ministry of Environment and Natural Resources (Secretaría de Medio Ambiente y Recursos Naturales; SEMARNAT) to JA. The sampling locality is the cenote Kan-Chin (Huhí, Yucatán), located at 20°40'11"N, 89°10'6"W. The voucher specimen was euthanized with MS-222 prior to preservation in accordance with recommended guidelines for the use of fishes in research (Nickum et al. 2004), fixed in a 10% formalin solution, and subsequently transferred to 70% ethanol for long-term storage in the Colección Nacional de Peces (CNPE) of the Instituto de Biología (IB) at the Universidad Nacional Autónoma de México (UNAM), where it has been catalogued and deposited (CNPEIBUNAM 23285). A tissue sample (muscle fragment) was taken prior to specimen fixation, preserved in 95% ethanol, and eventually cryopreserved at -80 °C. High-molecular genomic DNA was extracted using the phenol-chloroform protocol (Sambrook et al. 1989). The DNA was sheared by sonication with a Bioruptor pico of Diagenode and Minichiller. Sonication was performed using six cycles of alternating 30 s ultrasonic bursts and 30 s pauses in a 4 °C water bath. For library preparation we used a DNA sample of 200 ng which was quantified using a Qubit fluorometer (Invitrogen). Library preparation was carried out using the KAPA Biosystem Hyper Kit (Kapa, Biosystem Inc., Wilmington, MA). Fragmented DNA was ligated to custom, TruSeq-style dual-indexing adapters (Glenn et al. 2016). Fragments were size selected in a ~300–500 bp range which was enriched through PCR, purified and normalized. The Illumina NextSeq v2 300 cycle kit was used for sequencing paired-end 150 nucleotide reads at the Georgia Genomics Facility, University of Georgia, Athens, USA.

### ﻿Mitogenome assembly and annotation

The quality of the raw data was assessed with FastQC (Andrews, 2010). Good-quality sequences that did not contain ambiguous nucleotides and reads with average quality of 30Q were demultiplexed, trimmed and merged using Geneious Prime 2020.0.4 (https://www.geneious.com). Mitogenome assembly was conducted with MITObim v.1.9 (Hahn et al. 2013) using two reference mitogenomes from close relatives of *O.infernale* available in GenBank: *Ophisternoncandidum* (MT436449) and *Synbranchusmarmoratus* (AP004439). These reference mitogenomes were aligned in order to generate a consensus sequence for use during the annotation procedure. MitoFish MitoAnnotator (Iwasaki et al. 2013) and MITOS (Bernt et al. 2013) were used to identify and annotate protein-coding genes (PCGs), transfer RNAs (tRNAs), and ribosomal RNAs (rRNAs). The resultant annotated *O.infernale* mitochondrial genome was deposited in the GenBank database under accession number OM388306.

### ﻿Descriptive analyses

Nucleotide and amino acid composition, codon usage profiles of protein-coding genes (PCGs), Relative Synonymous Codon Usage (RSCU), and characterization the non-coding mtDNA control region (CR) were computed with mega X (Kumar et al. 2018). Nucleotide composition skewness was calculated with the formulas AT skew = (A − T)/(A + T) and GC skew = (G − C)/(G + C) (Perna and Kocher 1995). Prediction of tRNAs secondary structure was accomplished with tRNAScan-SE 2.0 (Chan and Lowe 2019) through the webserver http://lowelab.ucsc.edu/tRNAscan-SE/, using Infernal without HMM filter search mode and “vertebrate mitochondrial” as sequence source (Lowe and Chan 2016). Analysis and prediction of CR secondary structure in *O.infernale* was accomplished using the software ClustalW (Thompson et al. 2003) as implemented in mega X (Kumar et al. 2018) by comparison (via multiple sequence alignment) with reports of secondary CR structure from two other teleost fishes, namely *Sinipercachuatsi* (EU659698) (Zhao et al. 2006) and *Cyprinionsemiplotum* (MN603795) (Sharma et al. 2020).

### ﻿Comparative analyses

We investigated patterns of selection on PCGs on a mitogenomic scale and phylogenetic relationships among major synbranchiform lineages based on all mitogenomic comparative data for the group available on GenBank. To measure of the strength and mode of natural selection acting on PCGs, we estimated the ratio of non-synonymous (K_A_) to synonymous (K_S_) substitutions (K_A_/K_S_, also known as ω or *d*_N_/*d*_S_) using the HyPhy 2.5 package (Kosakovsky Pond et al. 2020) as implemented in mega X (Kumar et al. 2018) based on the newly assembled mitochondrial genome (*Ophisternoninfernale*, OM388306) and seven additional synbranchiform mitogenomes previously available in GenBank: *Ophisternoncandidum* (MT436449), *Synbranchusmarmoratus* (AP004439), *Monopterusalbus* (NC003192), *Mastacembelusarmatus* (NC023977), *Mastacembeluserythrotaenia* (NC035141), *Macrognathusaculeatus* (KT443991), and *Macrognathuspancalus* (NC032080). To compare patterns of selection between synbranchiform families, we conducted two separate K_A_/K_S_ analyses, one for synbranchids and one for mastacembelids. The taxonomic sampling for phylogenetic analyses included representatives of the synbranchiform families Synbranchidae and Mastacembelidae, as well as a representative of Indostomidae, a monogeneric family historically classified in the Gasterosteiformes on the basis of morphological evidence (Van Der Laan et al. 2014; Nelson et al. 2016) but more recently assigned to the Synbranchiformes in accordance to the results of molecular phylogenetic studies (Betancur-R. et al. 2013; Betancur-R et al. 2017). The lack of published mitochondrial genomes of fishes from the synbranchiform family Chaudhuriidae prevented us from including representatives of this taxon in our analyses. The ingroup consisted of the synbranchids *Ophisternoninfernale* (OM388306), *Ophisternoncandidum* (MT436449), *Synbranchusmarmoratus* (AP004439) and *Monopterusalbus* (NC003192), the mastacembelids *Mastacembelusarmatus* (NC023977), *Mastacembeluserythrotaenia* (NC035141), *Macrognathusaculeatus* (KT443991) and *Macrognathuspancalus* (NC032080), and the indostomid *Indostomusparadoxus* (NC004401). The outgroup consisted of representatives of close relatives of Synbranchiformes such as the anabantiforms *Channamicropeltes* (NC030542) and *Nandusnandus* (AP006809), and the gasterosteiforms *Gasterosteusaculeatus* (NC041244) and *Pungitiuspungitius* (NC011571); the last two included to test the phylogenetic position of *I.paradoxus* with respect to members of the Gasterosteiformes. The phylogeny was rooted at the viviparous brotula *Diplacanthopomabrachysoma* (AP004408). Phylogenetic relationships were inferred based on a concatenated alignment of all 13 PCGs. DNA sequence data from each PCG was independently aligned via multiple sequence alignment using the software MUSCLE (Edgar 2004) under default parameters via the “translation align” tool of the software Geneious Prime 2020.0.4 (https://www.geneious.com). The best-fit substitution model for each PCG was determined according to the corrected Akaike Information Criterion (AICc) with the software jModelTest2 (v. 2.1.10) (Darriba et al. 2012) under the following likelihood settings: number of substitution schemes = “3”; base frequencies = “+F”; rate variation = “+I and + G with nCat = 4”; base tree for likelihood calculations = “ML optimized”; and base tree search = “Best” (effectively evaluating among all 24 “classical” GTR-derived models). Individual alignments (*ATP6*=681 bp, *ATP8*=168 bp, *COX1*=1,539 bp, *COX2*=690 bp, *COX3*=783 bp, *CYTB*=1,137 bp, *NAD1*=975 bp, *NAD2*=1,053 bp, *NAD3*=348 bp, *NAD4*=1,380 bp, *NAD4L*=294 bp, *NAD5*=1,836 bp, and *NAD6*=525 bp) were subsequently concatenated using the software 2matrix (Salinas and Little 2014), yielding a data matrix totaling 11,409 aligned bp. Maximum Likelihood inference of phylogeny was carried out on the concatenated alignment partitioned by gene using the software RAxML-NG (v. 1.0.1) (Kozlov et al. 2019) through the CIPRES Science Gateway (Miller et al. 2010), with nodal support estimated by means of the bootstrap character resampling method (Felsenstein 1985) based on 1000 pseudoreplicates.

## ﻿Results and discussion

### ﻿Genome size and organization

**Figure 1.**
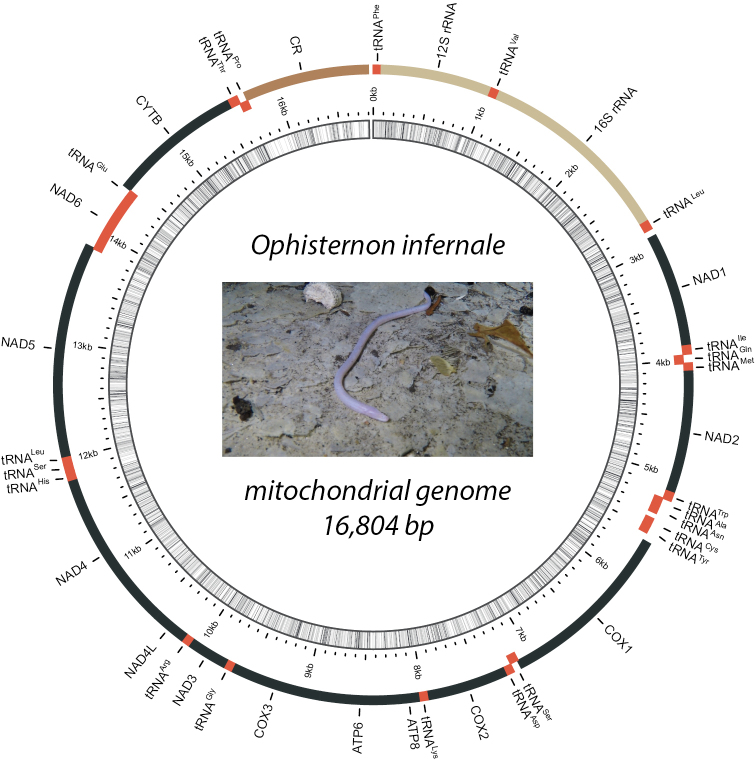
Annotated map of the mitochondrial circular genome of *O.infernale*. The outer ring corresponds to the H- (outermost) and L-strands, and depicts the location of PCGs (in black, except for *ND6* which is encoded in the L-strand and is portrayed in red), the non-coding control region (in dark brown), tRNAs (in red), and rRNAs (in light brown). The inner ring (black sliding window) denotes GC content along the genome. Live specimen photograph taken in the Cenote Kancabchen (Homún, Yucatán), courtesy of cave diver Erick Sosa.

The complete mitochondrial genome of *O.infernale* presented herein (GenBank accession number OM388306) is 16,804 bp in total length (Fig. 1; Table 1), a somewhat larger size than previously published synbranchiform mitogenomes, which range from 16,493 bp (in *M.erythrotaenia*) (or from 16,152 bp if considering the putative synbranchiform *I.paradoxus*) to 16,622 bp (in *M.albus*). Although the mitogenome of the synbranchid *S.marmoratus* reported in GenBank (AP004439) is considerably shorter (15,561 bp), this significant difference in length is actually due to it missing the *NAD1* gene (normally ~1,000 bp) as a result of reported technical difficulties during sequencing (Miya et al. 2003). The composition and general arrangement of mitochondrial genes in *O.infernale* is identical to that reported for other synbranchiforms (Li et al. 2016; Han et al. 2018; White et al. 2020) as well as for more distantly related teleosts (Miya et al. 2001, 2003; Satoh et al. 2016), and consists of a total of 37 genes divided into the following categories: 13 PCGs, 2 rRNAs, 22 tRNAs, and the non-coding control region (CR) (Fig. 1; Table 1). Twenty-eight genes (12 PCGs, 2 rRNAs, 14 tRNAs) plus CR are located on the H-strand, while the remaining nine genes (*NAD6* and 8 tRNAs) are located on the L-strand (Table 1); a configuration that corresponds to those of previously reported synbrachiform mitogenomes (Li et al. 2016; Han et al. 2018; White et al. 2020). The overall base composition of the *O.infernale* mitogenome is T=28.7%, A=31.6%, G=13.2%, and C=26.5%, which is fairly similar to those of other synbrachiform mitogenomes (Table 2). Nucleotide composition, however, is biased toward A+T (60.4%), with *O.infernale* displaying the highest values of this metric among the analyzed synbranchiforms. The mitogenome of *O.infernale* exhibits positive AT (0.046) and negative GC (-0.277) skewness, a general pattern shared with other species of the Synbranchiformes (Table 2).

**Table 1.** Mitochondrial genes and associated features of *O.infernale*. Intergenic space (IGS) described as intergenic (+) or overlapping nucleotides (–). AA = amino acid.

Locus	Type	One-letter code	Start	End	Length (bp)	Strand	# of AA	Anticodon	Start codon	Stop codon	IGS
*tRNA^Phe^*	tRNA	F	1	69	69	H		GAA			0
*12s rRNA*	rRNA		70	1017	948	H					0
*tRNA^Val^*	tRNA	V	1018	1091	74	H		TAC			0
*16s rRNA*	rRNA		1092	2766	1092	H					0
*tRNA-Leu*	tRNA	L	2767	2840	74	H		TAA			63
*NAD1*	Protein-coding		2904	3872	951	H	316		ATG	TAA	7
*tRNA^Ile^*	tRNA	I	3880	3949	70	H		GAT			8
*tRNA^Gln^*	tRNA	Q	3958	4028	71	L		TTG			–1
*tRNA^Met^*	tRNA	M	4028	4097	70	H		CAT			0
*NAD2*	Protein-coding		4098	5144	1047	H	337		ATG	AGA	–3
*tRNA^Trp^*	tRNA	W	5142	5211	70	H		TCA			1
*tRNA^Ala^*	tRNA	A	5213	5281	69	L		TGC			1
*tRNA^Asn^*	tRNA	N	5283	5355	73	L		GTT			53
*tRNA^Cys^*	tRNA	C	5409	5475	67	L		GCA			0
*tRNA^Tyr^*	tRNA	Y	5476	5542	67	L		GTA			1
*COX1*	Protein-coding		5544	7082	1539	H	489		GTG	AGA	–4
*tRNA^Ser^*	tRNA	S	7127	7197	71	L		TGA			2
*tRNA^Asp^*	tRNA	D	7200	7270	71	H		GTC			2
*COX2*	Protein-coding		7273	7963	691	H	225		ATG	T	0
*tRNA^Lys^*	tRNA	K	7964	8036	73	H		TTT			1
*ATP8*	Protein-coding		8038	8205	168	H	51		ATG	TAA	–8
*ATP6*	Protein-coding		8196	8878	683	H	223		ATG	TA	0
*COX3*	Protein-coding		8879	9662	784	H	249		ATG	T	0
*tRNA^Gly^*	tRNA	G	9663	9731	69	H		TCC			0
*NAD3*	Protein-coding		9732	10079	348	H	112		ATG	GAC	0
*tRNA^Arg^*	tRNA	R	10080	10148	69	H		TCG			0
*NAD4L*	Protein-coding		10149	10445	297	H	97		ATA	TAA	–5
*NAD4*	Protein-coding		10439	11819	1380	H	445		ATG	T	0
*tRNA^His^*	tRNA	H	11820	11888	69	H		GTG			0
*tRNA^Ser^*	tRNA	S	11889	11952	64	H		GCT			–1
*tRNA^Leu^*	tRNA	L	11952	12024	73	H		TAG			1
*NAD5*	Protein-coding		12026	13855	1830	H	598		ATG	TA	–2
*NAD6*	Protein-coding		13852	14373	522	L	172		ATG	T	1
*tRNA^Glu^*	tRNA	E	14375	14443	69	L		TTC			2
*CYTB*	Protein-coding		14446	15586	1141	H	369		ATG	T	0
*tRNA^Thr^*	tRNA	T	15587	15662	76	H		TGT			–1
*tRNA^Pro^*	tRNA	P	15662	15730	69	L		TGG			0
*D-loop*	Non-coding		15731	16804	1074	H					0

**Table 2.** Size and nucleotide composition of the complete synbranchiform mitochondrial genomes (and their concatenated PCGs) analyzed in this study. *NAD1 gene missing from published mitogenome.

Species	GenBank Accession #	Entire genome								Protein-coding genes			
		Length (bp)	A(%)	T(%)	C(%)	G(%)	AT(%)	AT skew	GC skew	Length (bp)	AT(%)	AT skew	GC skew
*Ophisternoninfernale*	OM388306	16804	31.6	28.7	26.5	13.2	60.4	0.046	-0.277	11449	60.1	-0.038	-0.348
*Ophisternoncandidum*	MT436449	16526	31.5	27.5	27.9	13.1	59	0.067	-0.36	11377	59.1	-0.015	-0.374
Synbranchusmarmoratus*	AP004439	15561	30.7	26.8	28.5	14	57.5	0.067	-0.341	10529	57.1	-0.027	-0.355
*Monopterusalbus*	NC003192	16622	28.9	27.2	29.4	14.5	56.1	0.03	-0.34	11430	54.9	-0.052	-0.356
*Mastacembelusarmatus*	NC023977	16487	29.1	25.3	30.9	14.7	54.4	0.069	-0.355	11404	53.1	-0.013	-0.381
*Mastacembeluserythrotaenia*	NC035141	16493	29	24.5	31.6	14.9	53.4	0.086	-0.357	11417	52.2	-0.003	-0.382
*Macrognathusaculeatus*	KT443991	16543	30	26.5	28.7	14.8	56.4	0.063	-0.322	11420	55.9	-0.014	-0.345
*Macrognathuspancalus*	NC032080	16549	29.7	26	29.6	14.7	55.7	0.664	-0.337	11420	54.9	-0.02	-0.363

### ﻿Protein-coding genes

**Figure 2.**
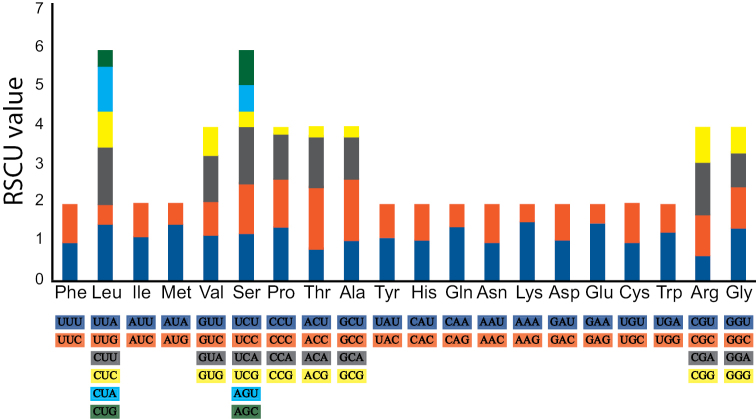
Results from analysis of Relative Synonymous Codon Usage (RSCU) of the mitochondrial genome of *O.infernale*. Codon families are plotted on the x-axis. The label for the 2, 4, or 6 codons that compose each family is shown in the boxes below the x-axis, and the colors correspond to those in the stacked columns. RSCU values are shown on the y-axis.

The 13 PCGs, altogether totaling 11,449 bp, correspond to 68.1% of the *O.infernale* mitogenome. These genes consist of seven regions that code for the subunits of the NADH dehydrogenase (ubiquinone) protein complex (*NAD1-6*, *NADL4*), three that code for the subunits of the enzyme cytochrome c oxidase (*COX1*-*3*), one that codes for the enzyme cytochrome b (*CYTB*), and two that code for the subunits 6 and 8 of the enzyme ATP synthase F_O_ (*ATP6*, *ATP8*). Except for *COX1* and *ND4L*, PCGs exhibit an ATG (Met) start codon­, which is the standard in eukaryotic systems (Kozak 1983). The start codon exhibited by *COX1* (GTG), however, is fairly common among vertebrates (Nwobodo et al. 2019). Conversely, an initiation-codon change from ATG (Met) to ATA (Ile) such as the one observed in *ND4L* is less common. Notably, of the synbranchiform mitogenomes analyzed, only that of *O.infernale* displays ATA as *ND4L* initiation codon. Most PCGs (10 out of 13) exhibit a TAA stop codon, which is a standard termination codon common in vertebrate mtDNA. However, of these 10 genes only three (*NAD1*, *NAD4L*, *ATP8*) display a complete codon (TAA), while the remaining seven (*ATP6*, *COX2*, *COX3*, *NAD4*, *NAD5*, *NAD6*, *CYTB*) contain an incomplete stop codon (either TA or T). Of the remaining three PCGs, *NAD2* and *COX1* have the stop codon AGA, while *NAD3* has the stop codon GAC (Table 2). PCGs in the mitogenome of *O.infernale* exhibit levels of A+T content (60.1%) comparable to–though slightly higher than–those of other synbranchiforms, which range from 53.1% in *M.armatus* to 59.1% in *O.candidum* (Table 2). In contrast to our findings for the entire mitogenome, AT-skews in PGCs across all synbranchiform mitogenomes analyzed exhibit negative values. Conversely, and in correspondence with our whole-mitogenome results, GC-skews in PGCs also exhibit negative values and highly similar across most analyzed synbranchiforms. A total of 3816 amino acids are encoded by PCGs in the mitogenome of *O.infernale*, with Leu (14.7%), Ser (9.4%), Thr (7.8%), and Pro (7.7%) being the most frequent, while Met (1.1%) being the least common. RSCU values represent the ratio between the observed usage frequency of one codon in a gene sample and the expected usage frequency in the synonymous codon family, given that all codons for the particular amino acid are used equally. The synonymous codons with RSCU values > 1.0 have positive codon usage bias and are defined as abundant codons, whereas those with RSCU values < 1.0 have negative codon usage bias and are defined as less-abundant codons (Gun et al. 2018). Results from RSCU analysis of PCGs in the mitogenome of *O.infernale* indicate that the most frequently used codons are ACC (1.59%), AAA (1.56%), TTA, ATA, and GAA (1.49%), which code for the amino acids Thr, Lys, Leu, Met, and Glu, respectively. On the other hand, codons encoding Prol (CCG, 0.16%), Thr (ACG, 0.2%), Ala (GCG, 0.23%), Ser (TCG, 0.39%), and Leu (CTG, 0.4%; TTA, 0.48%) are the least frequent (Fig. 2; Table 3).

**Table 3.** Results from the Relative Synonymous Codon Usage (RSCU) analysis for the PCGs of the mitochondrial genome of *O.infernale*.

Amino acid	Codon	Number	Freq. (%)	RSCU	Amino acid	Codon	Number	Freq. (%)	RSCU
Phe	TTT	110	2.9	1.02	Ala	GCA	48	1.3	1.12
	TTC	105	2.8	0.98		GCG	10	0.3	0.23
Leu	TTA	139	3.6	1.49	Tyr	TAT	119	3.1	1.13
	TTG	45	1.2	0.48		TAC	92	2.4	0.87
	CTT	144	3.8	1.54	His	CAU	59	1.5	1.08
	CTC	85	2.2	0.91		CAC	50	1.3	0.92
	CTA	110	2.9	1.18	Gln	CAA	78	2	1.42
	CTG	37	1	0.4		CAG	32	0.8	0.58
Ile	ATT	134	3.5	1.17	Asn	AAT	101	2.6	1
	ATC	95	2.5	0.83		AAC	102	2.7	1
Met	ATA	119	3.1	1.49	Lys	AAA	78	2	1.56
	ATG	41	1.1	0.51		AAG	22	0.6	0.44
Val	GTT	31	0.8	1.18	Asp	GAT	34	0.9	1.1
	GTC	23	0.6	0.88		GAC	28	0.7	0.9
	GTA	32	0.8	1.22	Glu	GAA	50	1.3	1.49
	GTG	19	0.5	0.72		GAG	17	0.4	0.51
Ser	TCT	73	1.9	1.22	Cys	TGT	30	0.8	0.98
	TCC	80	2.1	1.34		TGC	31	0.8	1.02
	TCA	88	2.3	1.47	Trp	TGA	63	1.7	1.26
	TCG	23	0.6	0.39		TGG	37	1	0.74
	AGT	42	1.1	0.7	Arg	CGT	14	0.4	0.67
	AGC	52	1.4	0.87		CGC	22	0.6	1.06
Pro	CCT	104	2.7	1.42		CGA	28	0.7	1.35
	CCC	91	2.4	1.24		CGG	19	0.5	0.92
	CCA	86	2.3	1.17	Gly	GGT	44	1.2	1.35
	CCG	12	0.3	0.16		GGC	36	0.9	1.11
Thr	ACT	66	1.7	0.87		GGA	30	0.8	0.92
	ACC	120	3.1	1.59		GGG	20	0.5	0.62
	ACA	101	2.6	1.34	Stop	TAA	83	2.2	1.78
	ACG	15	0.4	0.2		TAG	39	1	0.84
Ala	GCT	45	1.2	1.05		AGA	37	1	0.8
	GCC	69	1.8	1.6		AGG	27	0.7	0.58

### ﻿Transfer and ribosomal RNAs

**Figure 3.**
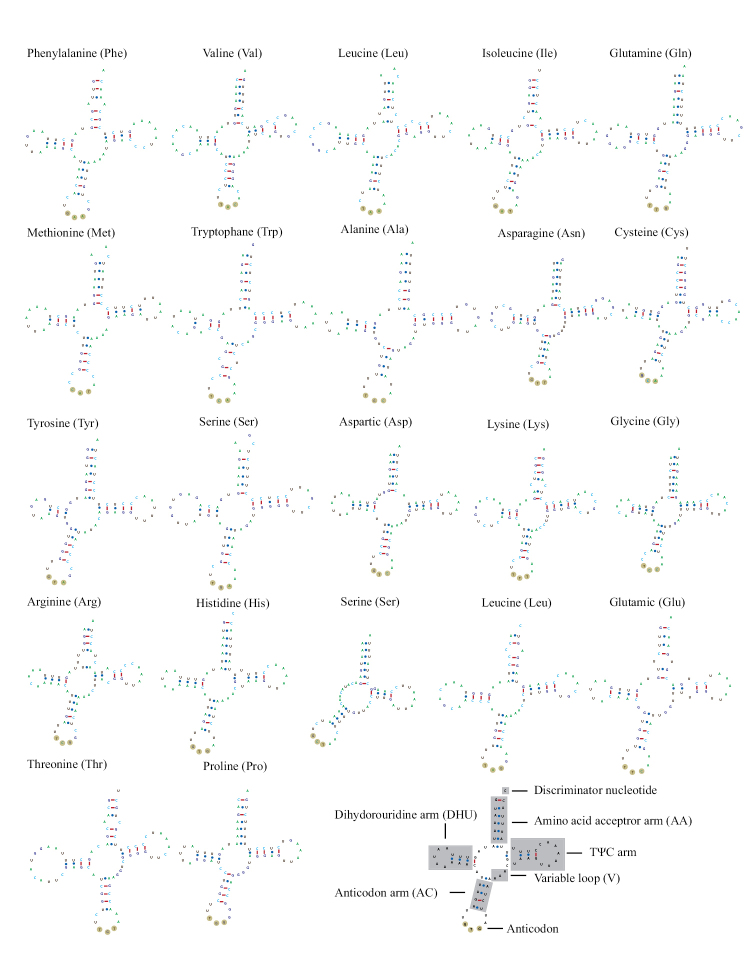
Secondary structure of the 22 tRNA genes of the mitochondrial genome of *O.infernale* predicted by tRNAScan-SE 2.0.

The mitogenome of *O.infernale* contains the typical 22 tRNAs usually documented for mitogenomes of other teleosts and vertebrates (Lee et al. 1995; Díaz-Jaimes et al. 2016; Satoh et al. 2016; Nwobodo et al. 2019; White et al. 2020). The genomic organization of tRNAs in *O.infernale* is identical to that reported for *O.candidum* (White et al. 2020) and other synbranchids (Li et al. 2016; Han et al. 2018). Altogether, tRNAs total 1547 bp, with individual ones ranging from 64 bp (tRNA^Ser^) to 76 bp (tRNA^Thr^) (Table 1). Fourteen tRNAs are encoded in the H-strand, while the remaining eight in the L-strand (Fig. 1; Table 1). Twenty-one of the 22 tRNAs fold into the canonical cloverleaf secondary structure that consists of four domains (AA stem, D arm, AC arm, and T arm) and a variable loop (Fig. 3). Notably, the tRNA^Ser^ (11889–11952) exhibits an unusual structure in which the D arm is missing. Although any change in tRNA secondary structure could potentially alter its amino acid recognition capability (Nwobodo et al. 2019), it has been shown that loss of the D arm does not necessarily imply reduced functionality; in fact, almost all tRNAs^Ser^ for AGY/N codons lack the D arm, and truncated tRNAs appear to have been compensated for by several interacting factors (Watanabe et al. 2014). Furthermore, among fishes, loss of the tRNA^Ser^ D arm is not unique to *O.infernale*, for it has been reported in several species, including chondrichthyans such as *Chiloscylliumgriseum* (Chen et al. 2013), *Triaenodonobesus* (Chen et al. 2016), and *Cephalloscylliumumbratile* (Zhu et al. 2017), as well as teleosts such as *Oreochromisandersonii* and *O.macrochir* (Bbole et al. 2018). Although most tRNAs present the canonical 7-bp T loop, nonstandard T-loop lengths were observed in tRNA^Met^ (6 bp), tRNA^Phe^ (8 bp), and tRNA^Ser^ (9 bp). Other deviations from the traditional tRNA secondary structure that could affect functionality is the presence of extra loops. The tRNA^Arg^ (10080–10148) in the mitogenome of *O.infernale* exhibits a loop at the base of the AA stem, thus potentially affecting aminoacylation. The nucleotide composition in the tRNAs of the *O.infernale* mitogenome is T=29%, A=31.4%, G=20.2%, and C=19.3%. The genes that code for the mitochondrial *12S* and *16S* rRNA subunits in *O.infernale* are 948 bp and 1092 bp long, respectively, and are located on the H-strand separated by the tRNA^Val^, just like in most teleost fishes (Lee et al. 1995; Satoh et al. 2016).

### ﻿Non-coding regions

**Figure 4.**
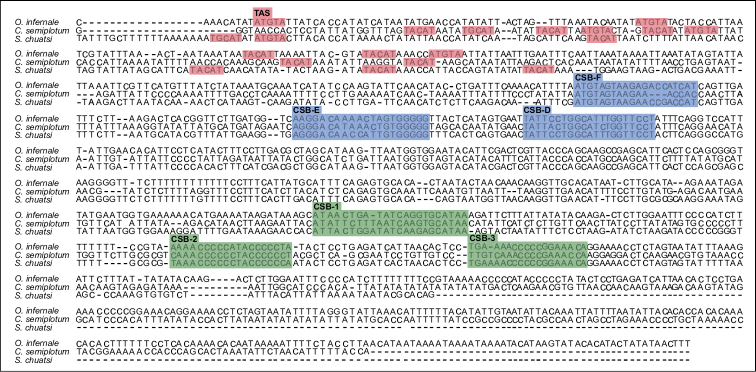
Comparison (multiple sequence alignment) of the mtDNA control region of *O.infernale* with those of fellow teleosts *Sinipercachuatsi* and *Cyprinionsemiplotum*. The alignment displays the three canonical domains distinguished by Termination Associated Sequences (TAS) of the upstream hypervariable region (in red), central conserved domain blocks (CSB-F, CSB-E, CSB-D) (in blue), and conserved sequence blocks of the downstream hypervariable region (CSB-1, CSB-2 and CSB-3) (in green).

The mtDNA control region of *O.infernale* is 1074 bp long (15731–16804), encoded in the H-strand, and flanked by tRNA^Pro^ and tRNA^Phe^ at the 5' and 3' ends, respectively (Fig. 1; Table 1), which is consistent with our understanding of mitogenome structure and organization in fishes (Lee et al. 1995; Rasmussen and Arnason 1999; Satoh et al. 2016). *Ophisternoninfernale*CR nucleotide composition is T=33.1%, A=36.7%, G=10.9%, and C=19.3%, with A+T content (69.8%) larger than that of the entire mitogenome but similar to that of other fishes including synbranchids (Li et al. 2016; Han et al. 2018). Like in other fishes, CR in *O.infernale* is divided into three domains: a central conserved domain flanked and two hypervariable domains (upstream and downstream). Three conserved sequence blocks (CSBs) were detected at the central conserved domain (CSB-F, CSB-E, CSB-D) as well as at the downstream hypervariable region (CSB1, CSB2, CSB3) (Fig. 4). Although additional CBSs have been identified for the central conserved domain (CSB-B, CSB-C) in mammals (Southern et al. 1988), the three identified herein for *O.infernale* are those commonly found in fishes (Broughton and Dowling 1994; Chen et al. 2012). The upstream hypervariable domain in the CR of *O.infernale* has a length of 256 bp and includes two copies of the motif TACAT and three copies of palindromic motif ATGTA. A change in the motif sequence (TGCAT) was observed in *C.semiplotum* and *S.chuatsi* but not in *O.infernale*. Compared to those from the central conserved domain, CSBs in the downstream hypervariable domain displayed larger variation across the three fish species compared. Notably, CSB2 and CSB3 were slightly more conserved than CSB1, a pattern that has been reported for other fishes (Chen et al. 2012).

### ﻿Patterns of selection on PCGs

**Figure 5.**
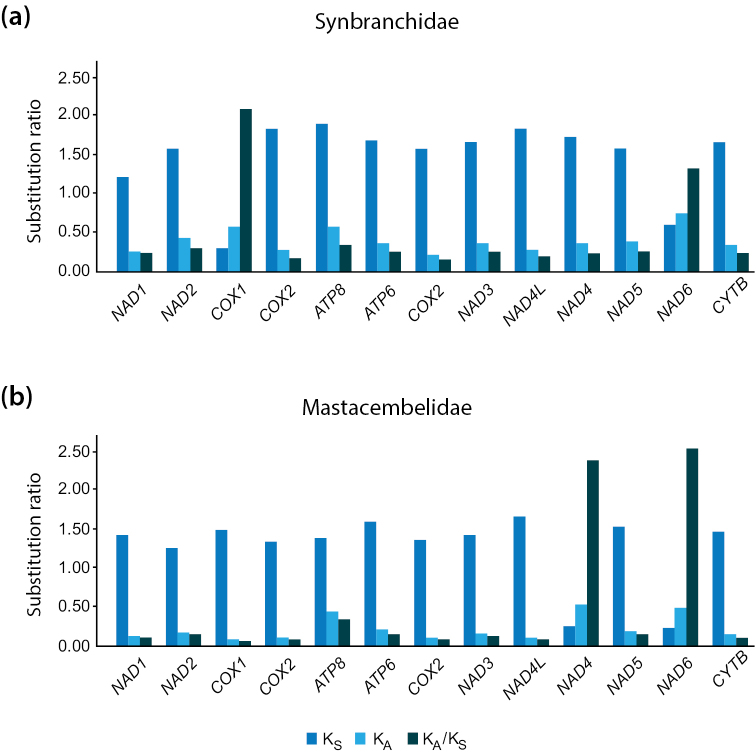
Patterns of selection in mtDNA PCGs of synbranchiform fishes. Results from K_A_/K_S_ ratio analysis on mitochondrial PCGs (x-axis) in synbranchiform fishes of the families Synbranchidae (**a**) and Mastacembelidae (**b**).

Results from K_A_/K_S_ analyses (Fig. 5) indicate that most mtDNA PCGs in synbranchiform fishes have evolved under strong purifying selection (K_A_/K_S_ <<1), preventing major structural and functional protein changes. Exceptions to this general pattern were observed for *COX1* and *NAD6* in synbranchids (Fig. 5a) and for *NAD4* and *NAD6* in mastacembelids (Fig. 5b), where significant signals of positive selection were detected. Studies in different groups of animals, including cephalopods (Almeida et al. 2015), rodents (Tomasco and Lessa 2011), and humans (DeHaan et al. 2004), have linked amino acid replacements in *NAD6* to adaptive selection to hypoxic conditions. Because numerous synbranchiform species are known to be fossorial and to inhabit low-oxygen waters, the observed signature of positive selection in *NAD6* might be related to adaptation to decreased oxygen availability. Notably, a recent comparative mitogenomic study of the African tilapias *Oreocrhomisandersonii* and *O.macrochir* similarly uncovered a pattern of positive selection in *NAD6* suggestive of adaptation in response to changing environments (Bbole et al. 2018). In contrast to the pattern observed for *NAD6*, selection in *COX1* and *NAD4* is completely conflicting between synbranchiform families. While in mastacembelids *COX1*–like most mitochondrial genes–has evolved under purifying selection (K_A_/K_S_ <1), the opposite happens in synbranchids. Although speculative at this point, the fact that half of our synbranchid dataset consists of troglomorphic cave-dwelling species (*O.infernale* and *O.candidum*) (vs. none in the mastacembelid dataset) could explain the observed differences in *COX1* selection patterns. Compared to surface waters, subterranean waters such as those of karst environments that harbor populations of *O.infernale* and *O.candidum* (in Mexico and Australia, respectively) contain low dissolved oxygen (Huppop 2000). Because of its role in aerobic metabolism, *COX1* might therefore be a target of directional selection promoting the evolution of more metabolically efficient variants in hypogean lineages (Boggs and Gross 2021). In contrast, the observed conflicting patterns of selection in *NAD4*–another gene involved in cellular respiration–between mastacembelids (positive) and synbranchids (purifying), do not seem to be readily explained by ecological differences related to cave life.

### ﻿Phylogeny and systematics of synbranchiform fishes

**Figure 6.**
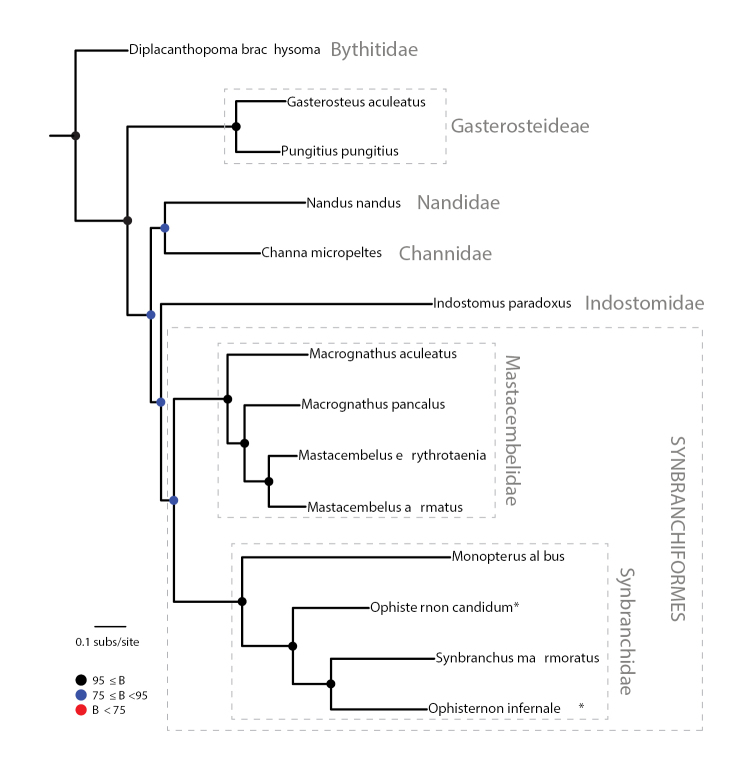
Phylogenetic relationships of major synbranchiform lineages. Molecular phylogeny based on comparative mitochondrial PCGs from relevant available mitogenomes and the newly generated herein for *O.infernale*. Troglobitic cave-dwelling species are marked with an asterisk to distinguish them from surface-dwelling ones. Outgroup taxa not shown. Colored circles on nodes indicate degree of clade support as determined by bootstrap values.

Our understanding of phylogenetic relationships in synbranchiform fishes is incipient compared to that of other teleost groups. Despite the fact that for the past two decades molecular systematics has been routinely employed to refine and update the classification of fishes and our knowledge of their evolutionary history (Betancur-R et al. 2017), a comprehensive molecular phylogeny of the Synbranchiformes has yet to be proposed. Apart from a phylogenetic study focused on Central American synbranchids (Perdices et al. 2005), no studies have investigated synbranchiform relationships using comparative DNA sequence data. Surprisingly, recent phylogenetic studies focused on higher-level relationships among major lineages of bony fishes (Betancur-R. et al. 2013; Betancur-R et al. 2017) resulted in the reassignment of armored sticklebacks (family Indostomidae, traditionally placed in the suborder Gasterosteoidei, order Scorpaeniformes) to the order Synbranchiformes. Although the classification of indostomids as gasterosteoids had been previously questioned on the basis of mitogenomic evidence (Miya et al. 2003, 2005; Kawahara et al. 2008), it was not until the phylogenetic classification of Betancur et al. (2013, 2017) that the family Indostomidae was transferred to the order Synbranchiformes. This proposal, however, was not adopted by the most authoritative contemporary standard references of fish systematics (Van Der Laan et al. 2014; Nelson et al. 2016), on the grounds of lack of morphological support and the need for further corroboration. It should be noted that previous molecular phylogenetic studies that cast doubt on the traditional placement of indostomids, whether based on “legacy” markers (Betancur-R. et al. 2013; Betancur-R et al. 2017) or complete mitochondrial genomes (Miya et al. 2003, 2005; Kawahara et al. 2008), relied on a very limited representation of synbranchiform diversity. In contrast, our phylogenetic analysis used mitogenomic data from a comparatively larger taxon sampling that included eight synbranchiform species from five genera (*Ophisternon*, *Synbranchus*, *Monopterus*, *Mastacembelus*, and *Macrognathus*) and two families (Synbranchidae, Mastacembelidae). Notably, our phylogenetic results (Fig. 6) corroborate the notion that indostomids are more closely related to synbranchiforms than to gasterosteoids. Nevertheless, contrary to the findings of studies that have recently challenged the traditional classification of indostomids with respect to synbranchiforms (Kawahara et al. 2008; Betancur-R. et al. 2013; Betancur-R et al. 2017), our inferred phylogenetic placement of *Indostomus* does not render Synbranchiformes paraphyletic. With the caveat that our sampling of synbranchiforms and closely related lineages is only partial, our results imply that indostomids are in fact the sister lineage of the order Synbranchiformes. While this phylogenetic pattern (topology) might be considered sufficient for lumping indostomids with synbranchiforms, examination of relative branch lengths (Fig. 6) suggests that *Indostomus* is indeed a highly divergent lineage. In order to acknowledge their genetic and morphological (Britz and Johnson 2002) distinctiveness, indostomids may in fact warrant an order of their own. Within Synbranchiformes, our results remarkably do not support the monophyly of the synbranchid genus *Ophisternon*, for *O.infernale* is resolved as more closely related to *Synbranchusmarmoratus* than to *O.candidum* (Fig. 6). While at first sight this novel finding of a sister-group relationship between *O.infernale* and *S.marmoratus* is certainly unexpected, this hypothesis might not be that far-fetched from a biogeographic perspective, and when considering both the striking external morphological similarity between the two genera and the taxonomic ambiguities surrounding the classificatory history of the group (Rosen and Greenwood 1976). *Synbranchus* is restricted to the New World and comprises three species: *S.marmoratus* (Central and South America), *S.madeirae* (Madeira River basin, Bolivia), and *S.lampreia* (Pará, Brazil). *Ophisternon* as currently delimited exhibits an essentially Gondwanan distribution, with six valid species distributed in Middle America (*O.infernale*, *O.aenigmaticum*), Australia (*O.candidum*, *O.gutturale*), South Asia and Western Pacific (*O.bengalense*), and West Africa (*O.afrum*). Assuming that Gondwanan drift vicariance is the main process responsible for the present-day globally disjunct distribution of the genus (Rosen 1975), the split between the Mexican-endemic *O.infernale* and the West Australian-endemic *O.candidum* should be at least as old as the Middle Jurassic separation of Eastern Gondwana (Antarctica, Madagascar, India, and Australia) from Western Gondwana (South America and Africa), dated at ca 165 Ma (McLoughlin 2001). From this it follows that the split between *Ophisternon* and *Synbranchus* should be even older. Notably, the only phylogenetic study that has investigated divergence times via molecular dating in a group of synbranchiforms (Perdices et al. 2005) estimated a comparatively much younger age (< 20 Ma) for the split between *Ophisternon (aenigmaticum)* and *Synbranchus (marmoratus)*. Although marine dispersal and extinction could be invoked in an attempt to reconcile biogeographic patterns with our admittedly limited knowledge of the timescale of synbranchiform diversification, the paraphyly of *Ophisternon* remains problematic. Our phylogenetic results coupled with the abovementioned estimates of synbranchid divergence times (Perdices et al. 2005) lead us to hypothesize that perhaps New World species of *Ophisternon* (*O.infernale* and *O.aenigmaticum*) are in fact more closely related to *Synbranchus* species than to the remaining *Ophisternon* species. As such, New World species of *Ophisternon* would have to be transferred to the genus *Synbranchus*. This phylogenetic scenario is also compatible with a likely very recent origin of the cave-dwelling *O.infernale*. Although there is virtually no information regarding the timing of origin and colonization of the fishes that inhabit the cenotes and submerged caves of the YP karstic aquifer (Arroyave et al. 2021), these aquatic habitats are supposed to be extremely young, effectively established not before 20,000 years ago, at the end of the last glacial maximum in the Northern Hemisphere, when rising sea levels eventually resulted in the flooding of karstic sinkholes and dry caves (Coke IV 2019). Such a recent origin for *O.infernale* is certainly much easier to explain as a result of speciation from a fellow New World lineage, such as *O.aenigmaticum* or *S.marmoratus*. Regardless of the appeal and feasibility of these hypotheses concerning the systematics of New World *Ophisternon* in general and the origins of *O.infernale* in particular, our phylogenetic findings and their interpretation need to be taken with caution because of their absolute reliance on mtDNA only. It is well known that the mitochondrial genome is effectively a single locus (Avise 2012), that individual gene and species trees are not always congruent (Maddison 1997), and that nuclear and mtDNA inheritance patterns are not always congruent either (Funk and Omland 2003). Notwithstanding these limitations, our results emphasize the pressing need for a comprehensive systematic and biogeographic study of synbranchiform fishes, ideally based on genome-wide sequence data.

## ﻿Conclusions

The first complete annotated mitochondrial genome of *O.infernale*, herein reported, exhibits an organization and arrangement similar to that of other synbranchiform fishes as well as of more distantly related teleosts. Based on our comparative mitogenomic dataset, most mitochondrial PCGs in synbranchiforms appear to have evolved under strong purifying selection, which has prevented major structural and functional protein changes. The few instances of mtDNA PCGs under positive selection might be related to adaptation to decreased oxygen availability and the evolution of more metabolically efficient variants in hypogean synbranchiform lineages. Phylogenetic analysis of mtDNA comparative data from synbranchiforms and closely related taxa (including the indostomid *Indostomusparadoxus*) corroborate the notion that indostomids are more closely related to synbranchiforms than to gasterosteoids, but without rendering the former paraphyletic. Our phylogenetic results also suggest that New World species of *Ophisternon* might be more closely related to *Synbranchus* than to the remaining *Ophisternon* species. This novel phylogenetic hypothesis, however, should be further tested in the context of a comprehensive systematic study of the group.
